# Association between dietary fiber to carbohydrate ratio and risk of dental caries in diabetic patients: an analysis of the National Health and Nutrition Examination Survey 2015–2020

**DOI:** 10.3389/fnut.2024.1440306

**Published:** 2024-07-04

**Authors:** Xue Liang, Hongbing Lu, Ping Lin, Xiaojing Huang

**Affiliations:** ^1^School and Hospital of Stomatology, Fujian Medical University, Fuzhou, Fujian, China; ^2^Fujian Key Laboratory of Oral Diseases, Fujian Medical University, Fuzhou, Fujian, China; ^3^Institute of Stomatology, Fujian Medical University, Fuzhou, China; ^4^Department of Stomatology, Nanping First Affiliated Hospital, Fujian Medical University, Nanping, Fujian, China

**Keywords:** dental caries, diabetes mellitus, fiber to carbohydrate ratio, diet, NHANES

## Abstract

**Aim:**

People with diabetes mellitus have a higher risk of dental caries than the general population. Diet is one of the most important factors affecting the risk of dental caries. This study aimed to evaluate the effect of dietary fiber to carbohydrate ratio (FCR) on the risk of dental caries in diabetic patients.

**Methods:**

Data of this cross-sectional study were extracted from the 2015–2020 cycle of the National Health and Nutrition Examination Survey (NHANES) database. FCR levels were divided into two categories based on the median (0.13). The outcomes were untreated dental caries and dental caries experience. The associations of FCR with untreated dental caries and dental caries experience were assessed using multivariable logistic regression analysis and reported as odds ratio (OR) and 95% confidence interval (CI). Stratified analyses were performed according to age (<65 and ≥ 65 years), gender (female and male), hypertension (yes and no), and the ratio of family income to poverty (PIR, <1 and ≥ 1).

**Results:**

A total of 2,412 patients diagnosed with diabetes were included, of whom 728 (30.18%) had untreated dental caries and 2,104 (87.23%) had dental caries experience. Patients with FCR ≥0.13 were correlated with lower odds of untreated dental caries (OR = 0.72, 95%CI: 0.52–0.99) and dental caries experience (OR = 0.63, 95%CI: 0.42–0.93) compared to patients with FCR <0.13. Stratified analyses demonstrated that patients with FCR ≥0.13 were found to be related to lower odds of untreated dental caries in those aged <65 years (OR = 0.64, 95%CI: 0.42–0.97), female (OR = 0.57, 95%CI: 0.35–0.93), with hypertension (OR = 0.66, 95%CI: 0.45–0.96), and PIR ≥1 (OR = 0.64, 95%CI: 0.42–0.99). Similar results to untreated dental caries were observed in the analysis of dental caries experience (*p* < 0.05).

**Conclusion:**

High levels of FCR may be associated with a lower risk of dental caries in patients with diabetes. Increasing the proportion of dietary fiber intake among diabetic patients may help prevent the occurrence of dental caries.

## Introduction

Dental caries is formed by the localized destruction of the hard tissues of the teeth by acidic by-products produced by bacterial fermentation of dietary carbohydrates (1). Symptoms of dental caries include pain, infection, and impaired esthetics, which not only affects oral health but also negatively impacts an individual’s quality of life (2, 3). Diabetes mellitus is a common and major chronic disease affecting human health and it aggravates oral complications such as periodontal disease and dental caries (4). Epidemiologic studies have shown that the prevalence of dental caries is significantly higher in diabetic patients than in non-diabetic patients (5, 6). Moreover, the presence of dental caries leads to a higher risk of heart failure in patients with diabetes (7). Therefore, identifying modifiable factors associated with dental caries is important to reduce the risk of dental caries and reduce the burden of disease in diabetic patients.

Dental caries begins with microbial changes in complex biofilms and is influenced by salivary flow and composition, fluoride exposure, dietary sugar consumption, and preventive behaviors (cleaning teeth) (1). Diet is an important modifiable influence in the development of diabetes and dental caries (8, 9). Carbohydrate intake, especially sugar, is an important risk factor for the development of dental caries (9, 10). Low dietary fiber intake may also be associated with an increased risk of dental caries (11, 12). The balance of carbohydrates and dietary fiber intake, the carbohydrate to dietary fiber ratio (CFR) or the dietary fiber to carbohydrate ratio (FCR), is a simple and practical indicator for assessing a healthy diet (13–16). Previous studies have found that CFR is significantly related to metabolic syndrome risk and metabolic risk factors (16–18). However, the effect of CFR or FCR on the risk of dental caries in diabetic patients is unclear. Thus, this study aimed to investigate the association between CFR or FCR and dental caries risk in diabetic patients. This may provide some basis for dietary prevention and control strategies for people at high risk of dental caries.

## Methods

### Study design and patients

Data for this cross-sectional study were extracted from three cycles of the National Health and Nutrition Examination Survey (NHANES), 2015–2016, 2017–2018, and 2019–2020. The NHANES is a nationally representative, cross-sectional, continuous survey conducted by the Centers for Disease Control and Prevention (CDC) to assess the nutritional and health status of the United States non-institutionalized population (19). NHANES collects data through interviews, which include demographic, socioeconomic, dietary, and health-related questions, and physical examinations, which include medical, dental, and physiological measurements, as well as laboratory tests. Because of some changes in the assessment of coronal caries in the NHANES database from 2015 (e.g., filled surfaces were assessed by restoration type) (20), only data from 2015 onward (2015–2016, 2017–2018, and 2019–2020) were included in this study. Participants included in this study were (1) aged ≥18 years; (2) diagnosed with diabetes mellitus; and (3) evaluated for dental caries. The exclusion criteria were as follows: (1) patients with missing information on dietary carbohydrate and fiber; (2) patients with unusually low or high total energy intake (<500 kcal/day or > 5,000 kcal/day for female, <500 kcal/day or > 8,000 kcal/day for male); and (3) patients with missing glycated hemoglobin (HbA1c) data. Diabetes mellitus was determined by self-reported diagnosis, use of insulin or oral hypoglycemic medication, HbA1c level ≥ 6.5%, or fasting blood glucose level ≥ 7.0 mmol/L (21). Protocols of NHANES were approved by the National Center for Health Statistics (NCHS) Ethics Review Board, and all participants signed informed consent. This study was waived by the Institutional Review Board of Fujian Medical University Hospital because the data were publicly available and de-identified.

### Outcomes

The outcomes of this study were presence of untreated dental caries and dental caries experience. Untreated dental caries was defined as the presence of untreated dental caries in at least one tooth. Dental caries experience was defined as the presence of at least one tooth decayed, missing, or filled due to dental caries (22). All oral health examination in the NHANES cycle were performed by trained and calibrated dentists at the mobile examination center (MEC). Each quadrant was air-dried after which 4 surfaces (lingual, buccal, mesial, distal) of anterior teeth, and 5 surfaces (occlusal, lingual, buccal, mesial, distal) of first and second molars were examined using a mirror and explorer (23). The decayed, missing, filled teeth (DMFT) index was used to assess dental caries. For the “untreated dental caries” variable, the tooth codes “J” and “Z” indicate the presence of untreated dental caries. For the “dental caries experience” variable, the tooth codes “E, F, J, P, R, T, and Z” were used to calculate the DMFT, with DMFT ≥1 defined as the presence of dental caries experience (22).

### Dietary data and other variables

Dietary fiber and carbohydrate intakes were estimated by 24-h dietary recall interview. The dietary recall interview collects detailed information about all foods and beverages consumed in the past 24 h. The first dietary recall interview is collected in-person in the MEC and the second interview is collected by telephone 3 to 10 days later (24). Food consumption data were converted to United States Department of Agriculture (USDA) standardized reference codes, and food energy and 64 nutrients/food components from each food/beverage were calculated using the USDA’s Food and Nutrient Database for Dietary Studies (FNDDS) (25). Dietary fiber and carbohydrate intakes include diet and supplements. Since the dietary fiber intake of some participants was 0, this study calculated the dietary fiber to carbohydrate ratio (FCR). FCR was calculated as: FCR = dietary fiber (gm)/carbohydrate (gm).

Other variables were collected including age (<65 and ≥ 65 years), gender (female and male), race (Mexican American, non-Hispanic Black, non-Hispanic White, other Hispanic, and other race), education level (less than 9^th^ grade and more than 9^th^ grade), marital status (married and others), ratio of family income to poverty (PIR; <1, ≥1, and unknown), body mass index (BMI), physical activity (≤450 MET⋅min/week, >450 MET⋅min/week, and no activity), smoking status (yes and no), drinking status (<1 time/week, 1–4 times/week, 5–7 times/week, and unknown), hypertension (yes and no), dental floss/device (yes and no), last dental visit (within last year, >1 year but ≤5 years, and > 5 years/never/unknown), total energy intake, total sugars, and HbA1c level (<7% and ≥ 7%). Smoking status was determined by the question “Have you smoked at least 100 cigarettes in your entire life,” and participants who answered “yes” were considered smokers.

### Statistical analysis

All analyses were performed using the SAS 9.4 software (SAS Institute Inc., Cary, NC, USA). The characteristics of diabetic patients were presented based on untreated dental caries and dental caries experience, respectively. Continuous variables were expressed as mean and standard error (S.E) and categorical variables were described as frequency and percentage [*n* (%)]. The Student’s t test was used for comparisons of continuous variables, and the chi-square test was used for comparisons of categorical variables.

Missing value of covariates were interpolated using Random-forest multiple imputation method. Sensitivity analysis was performed before and after interpolation of missing data. Univariable logistic regression analysis was utilized to screen for confounders associated with untreated dental caries and dental caries experience, respectively. Variables with *p* < 0.05 in univariable logistic regression analysis were included as confounders in multivariable logistic regression analyses for adjustment. FCR was divided into 2 categories based on the median (<1.3 and ≥ 1.3). The associations of FCR with untreated dental caries and dental caries experience were assessed using univariable-and multivariable logistic regression analyses and expressed as odds ratio (OR) and corresponding 95% confidence interval (CI). In addition, the correlations of FCR with untreated dental caries and dental caries experience were stratified by age (<65 and ≥ 65 years), gender (female and male), hypertension (yes and no), and PIR (<1 and ≥ 1). Statistical significance was set at *p* < 0.05.

## Results

### Patient characteristics

**Figure 1:**
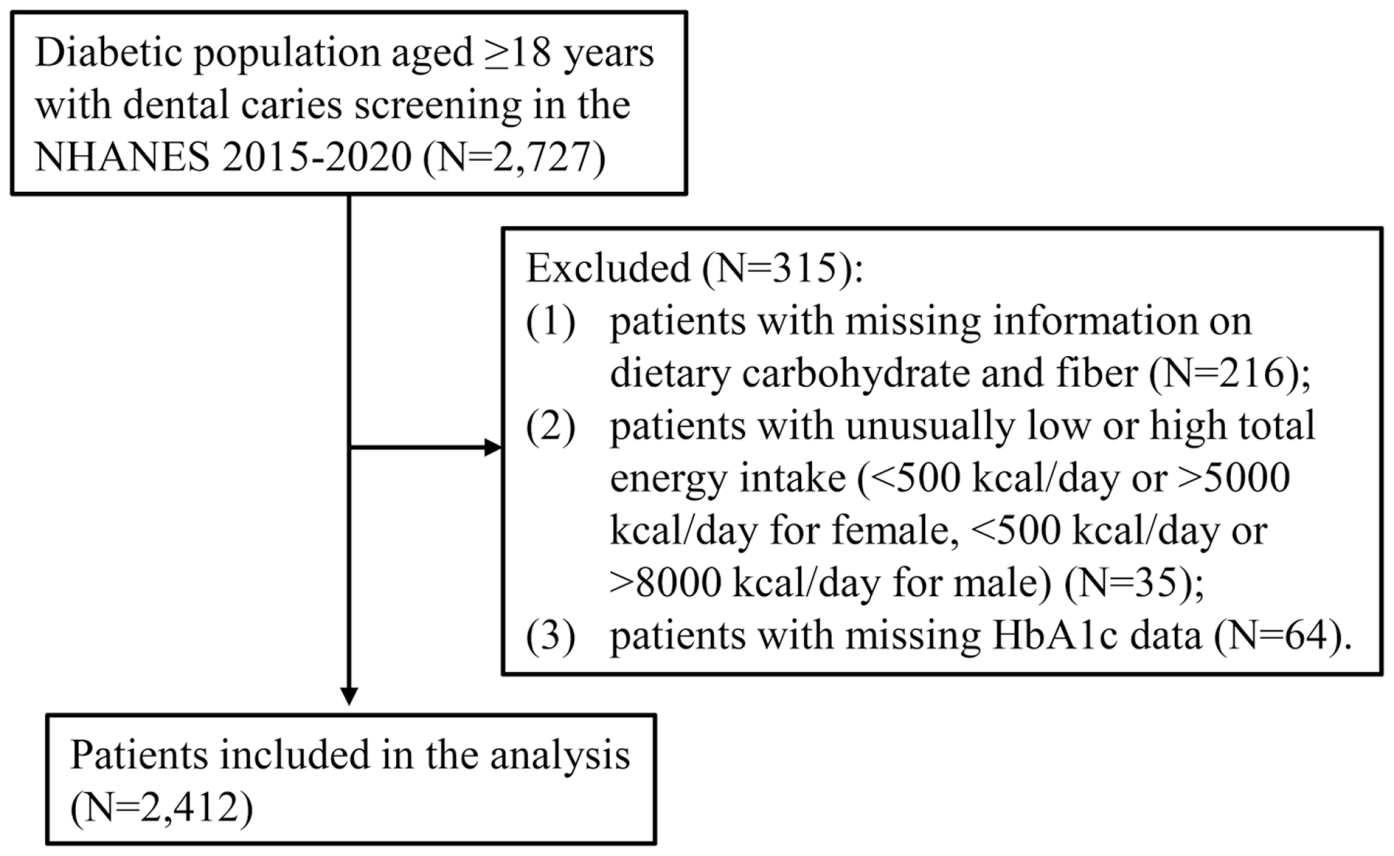
Flowchart for screening participants in this study.

A total of 2,727 diabetic patients who were screened for dental caries were identified from the 2015–2020 NHANES. After excluding 315 ineligible patients, 2,412 patients were included in the analysis (Figure 1). The characteristics of 2,412 diabetic patients were presented in Table 1. There were 1,402 (59.40%) patients younger than 65 years of age and 1,275 (53.31%) male patients. The mean (S.E) BMI of these patients was 33.08 (0.19) kg/m^2^. There were 1,132 (47.74%) patients who smoked cigarettes and 1,438 (50.14%) patients who consumed alcohol <1 time/week. There were 1,211 (57.48%) patients with dental visit within last year and 1,803 (79.72%) patients with dental care past year. The mean (S.E) total sugars intake was 95.87 (2.18) gm. The mean (S.E) dietary fiber intake and carbohydrate intake were 16.60 (0.40) gm and 228.10 (4.09) gm, respectively. The mean (S.E) FCR value was 0.15 (0.00). There were 728 (30.18%) patients with untreated dental caries and 2,104 (87.23%) patients with dental caries experience.

**Table 1:** Characteristics of diabetic patients based on untreated dental caries and dental caries experience.

Variables	Total (*n* = 2,412)	Untreated dental caries			Dental caries experience		
		No (*n* = 1,684)	Yes (*n* = 728)	*p*-value	No (*n* = 308)	Yes (*n* = 2,104)	*p*-value
Age, years, *n* (%)				0.003			<0.001
<65	1,402 (59.40)	928 (55.86)	474 (68.95)		256 (78.98)	1,146 (55.24)	
≥65	1,010 (40.60)	756 (44.14)	254 (31.05)		52 (21.02)	958 (44.76)	
Gender, *n* (%)				0.408			<0.001
Female	1,137 (46.69)	799 (46.00)	338 (48.54)		128 (35.15)	1,009 (49.13)	
Male	1,275 (53.31)	885 (54.00)	390 (51.46)		180 (64.85)	1,095 (50.87)	
Race, *n* (%)				<0.001			0.649
Mexican American	413 (10.31)	271 (9.11)	142 (13.56)		54 (9.76)	359 (10.43)	
Non-Hispanic Black	635 (13.33)	382 (11.03)	253 (19.58)		58 (10.69)	577 (13.90)	
Non-Hispanic White	742 (59.21)	570 (62.83)	172 (49.44)		107 (62.74)	635 (58.47)	
Other Hispanic	287 (6.70)	209 (6.59)	78 (6.97)		30 (6.02)	257 (6.84)	
Other Race	335 (10.44)	252 (10.44)	83 (10.45)		59 (10.80)	276 (10.37)	
Education level, *n* (%)				0.077			<0.001
Less than 9th Grade	317 (7.46)	221 (6.72)	96 (9.46)		21 (2.73)	296 (8.46)	
More than 9th Grade	2095 (92.54)	1,463 (93.28)	632 (90.54)		287 (97.27)	1808 (91.54)	
Marital status, *n* (%)				0.005			0.125
Married	1,406 (60.69)	1,024 (63.65)	382 (52.66)		210 (66.03)	1,196 (59.55)	
Others	1,006 (39.31)	660 (36.35)	346 (47.34)		98 (33.97)	908 (40.45)	
PIR, *n* (%)				<0.001			<0.001
<1	481 (14.59)	277 (11.13)	204 (23.93)		29 (5.72)	452 (16.47)	
≥1	1,668 (77.60)	1,233 (81.21)	435 (67.82)		259 (89.17)	1,409 (75.15)	
Unknown	263 (7.81)	174 (7.65)	89 (8.24)		20 (5.10)	243 (8.39)	
BMI, kg/m^2^, Mean (S.E)	33.08 (0.19)	32.94 (0.22)	33.44 (0.41)	0.302	33.78 (0.59)	32.93 (0.22)	0.204
Physical activity, *n* (%)				0.481			0.052
≤450 MET⋅min/week	244 (9.73)	177 (10.34)	67 (8.08)		31 (9.03)	213 (9.88)	
>450 MET⋅min/week	1,302 (57.43)	902 (58.06)	400 (55.72)		198 (65.96)	1,104 (55.62)	
No activity	866 (32.84)	605 (31.59)	261 (36.20)		79 (25.01)	787 (34.50)	
Smoking status, *n* (%)				0.209			<0.001
No	1,280 (52.26)	918 (53.35)	362 (49.28)		208 (70.42)	1,072 (48.40)	
Yes	1,132 (47.74)	766 (46.65)	366 (50.72)		100 (29.58)	1,032 (51.60)	
Drinking status, *n* (%)				0.082			0.010
<1 time/week	1,438 (50.14)	1,007 (49.64)	431 (51.49)		169 (43.75)	1,269 (51.50)	
1–4 times/week	206 (7.93)	146 (8.60)	60 (6.15)		43 (14.33)	163 (6.58)	
5–7 times/week	59 (2.68)	43 (3.25)	16 (1.16)		11 (3.33)	48 (2.55)	
Unknown	709 (39.24)	488 (38.51)	221 (41.21)		85 (38.59)	624 (39.38)	
Hypertension, *n* (%)				0.432			<0.001
No	292 (13.52)	209 (14.06)	83 (12.04)		68 (26.02)	224 (10.87)	
Yes	2,120 (86.48)	1,475 (85.94)	645 (87.96)		240 (73.98)	1880 (89.13)	
Dental floss/device, *n* (%)				0.032			0.093
No	936 (32.70)	631 (31.06)	305 (37.15)		75 (27.15)	861 (33.88)	
Yes	1,476 (67.30)	1,053 (68.94)	423 (62.85)		233 (72.85)	1,243 (66.12)	
Dental care past year, *n* (%)				<0.001			<0.001
Yes	1803 (79.72)	1,382 (86.05)	421 (62.61)		263 (88.90)	1,540 (77.78)	
No	609 (20.28)	302 (13.95)	307 (37.39)		45 (11.10)	564 (22.22)	
Last dental visit, *n* (%)				<0.001			0.004
Within last year	1,211 (57.48)	954 (64.76)	257 (37.78)		198 (66.91)	1,013 (55.48)	
>1 year but ≤5 years	718 (25.54)	450 (21.37)	268 (36.82)		71 (24.45)	647 (25.77)	
>5 years/never/unknown	483 (16.98)	280 (13.87)	203 (25.41)		39 (8.64)	444 (18.75)	
Dietary fiber, gm, Mean (S.E)	16.60 (0.40)	16.86 (0.46)	15.89 (0.74)	0.261	17.16 (0.54)	16.48 (0.49)	0.406
Carbohydrate, gm, Mean (S.E)	228.10 (4.09)	228.98 (5.02)	225.70 (6.49)	0.689	234.93 (6.64)	226.65 (4.97)	0.359
Total Energy intake, kcal, Mean (S.E)	1985.72 (38.92)	2015.58 (49.37)	1904.94 (42.83)	0.083	2103.66 (51.06)	1960.71 (46.02)	0.048
Total sugars, gm, Mean (S.E)	95.87 (2.18)	94.63 (2.40)	99.21 (4.93)	0.411	93.95 (4.38)	96.27 (2.76)	0.692
HbA1c level, *n* (%)				0.006			0.105
<7%	1,303 (59.89)	934 (62.05)	369 (54.05)		183 (65.42)	1,120 (58.72)	
≥7%	1,109 (40.11)	750 (37.95)	359 (45.95)		125 (34.58)	984 (41.28)	
FCR, Mean (S.E)	0.15 (0.00)	0.15 (0.00)	0.14 (0.01)	0.039	0.16 (0.01)	0.15 (0.00)	0.101
FCR level, *n* (%)				0.023			<0.001
<0.13	1,253 (49.80)	846 (47.04)	407 (57.27)		128 (36.32)	1,125 (52.66)	
≥0.13	1,159 (50.20)	838 (52.96)	321 (42.73)		180 (63.68)	979 (47.34)	

PIR, ratio of family income to poverty; BMI, body mass index; HbA1c, glycated hemoglobin; FCR, dietary fiber to carbohydrate ratio.

### Factors related to untreated dental caries and dental caries experience in diabetic patients

The factors may be related to untreated dental caries and dental caries experience were shown in Supplementary Table S1. The univariable logistic analysis found that older age, non-Hispanic White, high PIR, dental floss/device use, and dental visit within last year were associated with lower odds of untreated dental caries, whereas unmarried, HbA1c level ≥ 7%, and no dental care past year were correlated with higher odds of untreated dental caries (*p* < 0.05). For the analysis of dental caries experience, male, high education level, and high PIR were associated with lower odds of dental caries experience, while older age, smoking, drinking alcohol <1 time/week, hypertension, no dental care past year, and last dental visit more than 5 years were related to higher odds of dental caries experience (*p* < 0.05).

### Association of FCR with untreated dental caries and dental caries experience

Table 2 presents the correlations of FCR with untreated dental caries and dental caries experience in diabetic patients. Patients with FCR ≥0.13 were associated with lower odds of untreated dental caries (OR = 0.66, 95%CI: 0.46–0.96) and dental caries experience (OR = 0.51, 95%CI: 0.35–0.75) compared to patients with FCR <0.13. After controlling confounders, patients with FCR ≥0.13 were still correlated with lower odds of untreated dental caries (OR = 0.72, 95%CI: 0.52–0.99) and dental caries experience (OR = 0.63, 95%CI: 0.42–0.93) compared to patients with FCR <0.13. Sensitivity analysis showed that the results before and after interpolation of missing data were consistent (Supplementary Table S2).

**Table 2:** Association of FCR with untreated dental caries and dental caries experience in diabetic patients.

Analysis	Variables	Untreated dental caries		Dental caries experience	
		OR (95%CI)	*p*-value	OR (95%CI)	*p*-value
Univariable analysis	FCR <0.13	Ref		Ref	
	FCR ≥0.13	0.66 (0.46–0.96)	0.029	0.51 (0.35–0.75)	<0.001
Multivariable analysis	FCR <0.13	Ref		Ref	
	FCR ≥0.13	0.72 (0.52–0.99)	0.048	0.63 (0.42–0.93)	0.021

FCR, dietary fiber to carbohydrate ratio; OR, odds ratio; CI, confidence interval; Ref, reference; Multivariable logistic regression analysis adjusted for: (1) age, drinking status, race, marital status, PIR, dental floss/device use, Hba1c (untreated dental caries); (2) age, gender, education level, PIR, smoking status, hypertension, dental floss/device use (dental caries experience).

**Figure 2:**
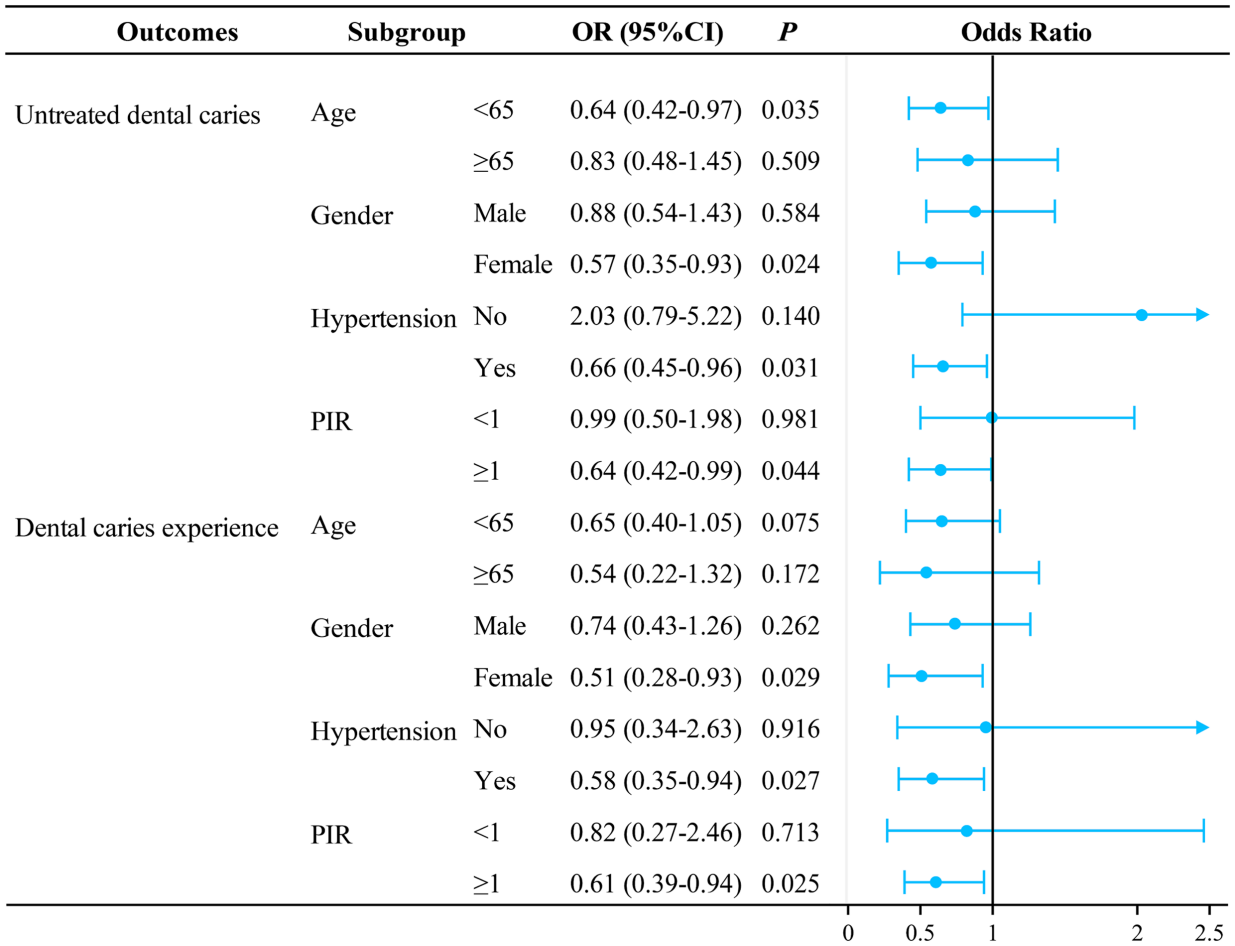
Stratified analysis of the association between FCR and untreated dental caries and dental caries experience in diabetic patients. All analyses were multivariable logistic regression analysis adjusted for: (1) age, drinking status, race, marital status, PIR, dental floss/device use, Hba1c (untreated dental caries); (2) age, gender, education level, PIR, smoking status, hypertension, dental floss/device use (dental caries experience). FCR, dietary fiber to carbohydrate ratio; PIR, ratio of family income to poverty; OR, odds ratio; CI, confidence interval; Ref, reference.

Figure 2 shows a stratified analysis of the association between FCR and untreated dental caries and dental caries experience. Patients with FCR ≥0.13 were found to be correlated to lower odds of untreated dental caries in those aged <65 years (OR = 0.64, 95%CI: 0.42–0.97), female (OR = 0.57, 95%CI: 0.35–0.93), with hypertension (OR = 0.66, 95%CI: 0.45–0.96), and PIR ≥1 (OR = 0.64, 95%CI: 0.42–0.99). For the analysis of dental caries experience, patients with FCR ≥0.13 were correlated with lower odds of dental caries experience among female (OR = 0.51, 95%CI: 0.28–0.93), those with hypertension (OR = 0.58, 95%CI: 0.35–0.94), and PIR ≥1 (OR = 0.61, 95%CI: 0.39–0.94).

## Discussion

This study explored the effect of dietary fiber to carbohydrate ratio (FCR) on the risk of dental caries in diabetic patients. The results demonstrated that patients with FCR ≥0.13 were correlated with lower odds of untreated dental caries and dental caries experience compared to those with FCR <0.13. In addition, the relationship between high FCR levels and lower odds of dental caries was only observed in those <65 years, female, with hypertension, and PIR ≥1.

The pathophysiologic manifestations of diabetes can cause lasting damage to multiple body systems. The presence of diabetes may also alter salivary composition and salivary flow rate, as well as increase oral mucosal lesions, leading to an increase in oral complications such as periodontal disease and dental caries (26). The occurrence of dental caries is influenced by a variety of biological and social factors such as gender, age, oral hygiene, genetic susceptibility, socioeconomic status, diet, and dental services (27–29). Diet is the most common and modifiable factor that plays an important role in the development and prevention of dental caries, of which carbohydrate intake is a significant risk factor (8, 9). Frequency of dietary habits and consumption of large amounts of fermentable carbohydrates are associated with the risk of dental caries (30). The present study investigated the relationship between FCR and dental caries risk in diabetic patients. The results indicated that patients with FCR ≥0.13 were related to lower odds of untreated dental caries and dental caries experience. The balance between total carbohydrates and dietary fiber is an important indicator of a healthy diet, i.e., at least 1 g of fiber per 10 g of carbohydrates (10:1) (15). The dietary fiber to carbohydrate ratio in our study was classified according to a median of 0.13, which suggested that patients with FCR values greater than 0.13 may have a healthier diet. The association between carbohydrate intake and dental caries risk may be related to the growth of oral microorganisms and carbohydrate metabolism leading to local acidification and disruption of dental mineralization homeostasis (31). The reduced dental caries risk associated with the intake of dietary fiber-rich vegetables and fruits may be related to the fact that these foods are rich in nutrients that limit the development of dental caries, such as isethionates and polyphenols (32). In addition, it is not possible to consume excessive amounts of the naturally occurring sugars in fruits and vegetables, and the active substances (antioxidants) that are widely available in fruits and vegetables reduce inflammation and improve endothelial function (33).

The association between FCR and dental caries risk may vary in different populations. Our results showed that patients with FCR ≥0.13 were found to be related to lower odds of untreated dental caries in those aged <65 years, female, with hypertension, and PIR ≥1. The incidence of untreated dental caries in different age groups has a major peak in young children, followed by a second, lower peak in adolescents and young adults (3). A trough occurs around the age of 40, after which the incidence rises gradually with age (3). The current study found a relationship between high FCR levels and lower risk of untreated dental caries only in patients aged <65 years. This may suggest the important of dietary interventions at a young age. Females generally show a higher prevalence of dental caries and severity of disease than males (34). This sex difference may be related to sex hormones, genetic factors, and dietary habits (34, 35). This may also explain the association between high FCR levels and lower risk of untreated caries observed only in females. Previous studies have confirmed the association between low socioeconomic status and high risk of caries/caries experience (36). This may be associated with the health knowledge and behaviors of populations at higher socioeconomic status, such as healthy dietary and dental cleaning habits, frequency and patterns of health service use (36). The association between hypertension and dental caries presented inconsistent results (37). The correlation between high FCR levels and lower risk of untreated dental caries was found only in patients with hypertension, which may require additional subsequent studies to explain this result.

This study evaluated the effect of FCR on the risk of dental caries in diabetic patients using a large sample of nationally representative NHANES data. FCR as a dietary indicator that can be easily changed in daily routine plays an important role in the prevention of dental caries risk in diabetic patients. Furthermore, several limitations of this study should be considered. First, the cross-sectional study design of this study could not confirm a causal association between FCR and dental caries risk. Second, although the dietary intake in our study included daily diets and supplements, this only reflects recent eating habits, and the effects of long-term dietary fiber and carbohydrate intake on dental caries risk need to be further explored. Third, some factors that may influence dental caries such as the use of fluoride toothpaste were not considered due to the limitations of data recording in the NHANES database.

## Conclusion

This study investigated the effect of the ratio of dietary fiber to carbohydrate intake on the risk of dental caries in diabetic patients. High levels of FCR may be associated with a lower risk of dental caries in patients with diabetes. Increasing the proportion of dietary fiber intake among diabetic patients may help prevent the occurrence of dental caries. However, the effects of long-term dietary fiber and carbohydrate intake on the risk of dental caries may need to be further explored.

## Data availability statement

Publicly available datasets were analyzed in this study. This data can be found at: NHANES database, https://wwwn.cdc.gov/nchs/nhanes/.

## Ethics statement

The requirement of ethical approval was waived by School and Hospital of Stomatology, Fujian Medical University for the studies involving humans because School and Hospital of Stomatology, Fujian Medical University. The studies were conducted in accordance with the local legislation and institutional requirements. The participants provided their written informed consent to participate in this study.

## Author contributions

XL: Conceptualization, Project administration, Supervision, Writing – original draft, Writing – review & editing. HL: Data curation, Formal analysis, Investigation, Methodology, Writing – review & editing. PL: Data curation, Formal analysis, Investigation, Methodology, Writing – review & editing. XH: Conceptualization, Project administration, Writing – original draft, Writing – review & editing.
