# Supplementary material for: Association between dietary fiber to carbohydrate ratio and risk of dental caries in diabetic patients: an analysis of the National Health and Nutrition Examination Survey 2015–2020
Source: Front Nutr. 2024 Jul 4;11:1440306. doi: 10.3389/fnut.2024.1440306 (PMC11254849; doi:10.3389/fnut.2024.1440306)
Supplement: Supplementary file 1 [file Table_1.DOCX]

**Supplement Table 1**. Univariable logistic regression analysis of factors related to untreated dental caries and dental caries experience in diabetic patients.

| **Variables** | **Untreated dental caries** | | **Dental caries experience** | |
| --- | --- | --- | --- | --- |
|  | **OR (95%CI)** | ***P*** | **OR (95%CI)** | ***P*** |
| Age |  |  |  |  |
| <65 years | Ref |  | Ref |  |
| ≥65 years | 0.57 (0.38-0.85) | 0.008 | 3.04 (1.77-5.23) | <0.001 |
| Gender |  |  |  |  |
| Female | Ref |  | Ref |  |
| Male | 0.90 (0.70-1.16) | 0.414 | 0.56 (0.39-0.80) | 0.002 |
| Race |  |  |  |  |
| Mexican American | Ref |  | Ref |  |
| Non-Hispanic Black | 1.19 (0.67-2.14) | 0.545 | 1.22 (0.71-2.09) | 0.471 |
| Non-Hispanic White | 0.53 (0.30-0.94) | 0.030 | 0.87 (0.57-1.34) | 0.520 |
| Other Hispanic | 0.71 (0.40-1.25) | 0.228 | 1.06 (0.60-1.88) | 0.828 |
| Other Race | 0.67 (0.36-1.26) | 0.209 | 0.90 (0.53-1.53) | 0.684 |
| Education level |  |  |  |  |
| Less Than 9th Grade | Ref |  | Ref |  |
| More than 9th Grade | 0.69 (0.44-1.08) | 0.099 | 0.30 (0.17-0.54) | <0.001 |
| Marital status |  |  |  |  |
| Married | Ref |  | Ref |  |
| Others | 1.57 (1.14-2.18) | 0.007 | 1.32 (0.91-1.91) | 0.135 |
| PIR |  |  |  |  |
| <1 | Ref |  | Ref |  |
| ≥1 | 0.39 (0.29-0.52) | <0.001 | 0.29 (0.17-0.50) | <0.001 |
| Unknown | 0.50 (0.32-0.79) | 0.004 | 0.57 (0.21-1.53) | 0.258 |
| BMI | 1.01 (0.99-1.03) | 0.293 | 0.98 (0.96-1.01) | 0.199 |
| Physical activity |  |  |  |  |
| No activity | 1.47 (0.71-3.02) | 0.289 | 1.26 (0.63-2.52) | 0.502 |
| ≤450 MET⋅min/week | Ref |  | Ref |  |
| >450 MET⋅min/week | 1.23 (0.71-2.12) | 0.447 | 0.77 (0.39-1.54) | 0.452 |
| Smoking status |  |  |  |  |
| No | Ref |  | Ref |  |
| Yes | 1.18 (0.91-1.52) | 0.209 | 2.54 (1.58-4.08) | <0.001 |
| Drinking status |  |  |  |  |
| <1 time/week | 1.45 (0.88-2.39) | 0.139 | 2.56 (1.59-4.14) | <0.001 |
| 1-4 times/week | Ref |  | Ref |  |
| 5-7 times/week | 0.50 (0.24-1.04) | 0.064 | 1.67 (0.47-5.94) | 0.420 |
| Unknown | 1.50 (0.83-2.70) | 0.174 | 2.22 (1.26-3.92) | 0.007 |
| HbA1c level |  |  |  |  |
| <7% | Ref |  | Ref |  |
| ≥7% | 1.39 (1.09-1.77) | 0.009 | 1.33 (0.93-1.90) | 0.116 |
| Hypertension |  |  |  |  |
| No | Ref |  | Ref |  |
| Yes | 1.20 (0.76-1.89) | 0.436 | 2.89 (1.69-4.94) | <0.001 |
| Dietary fiber | 0.99 (0.97-1.01) | 0.293 | 0.99 (0.98-1.01) | 0.414 |
| Carbohydrate | 1.00 (1.00-1.00) | 0.693 | 1.00 (1.00-1.00) | 0.358 |
| Total Energy intake | 1.00 (1.00-1.00) | 0.076 | 1.00 (1.00-1.00) | 0.059 |
| Total sugars | 1.00 (1.00-1.00) | 0.369 | 1.00 (1.00-1.00) | 0.696 |
| Dental care past year |  |  |  |  |
| No | Ref |  | Ref |  |
| Yes | 0.76 (0.59-0.99) | 0.042 | 0.73 (0.50-1.07) | 0.102 |
| Last dental visit |  |  |  |  |
| No | Ref |  | Ref |  |
| Yes | 3.68 (2.76-4.92) | <0.001 | 2.29 (1.43-3.67) | 0.001 |
| Last dental visit |  |  |  |  |
| Within last year | 0.34 (0.24-0.47) | <0.001 | 0.79 (0.50-1.23) | 0.289 |
| >1 year but ≤ 5 years | Ref |  | Ref |  |
| >5 years/never/unknown | 1.06 (0.73-1.56) | 0.746 | 2.06 (1.15-3.68) | 0.016 |

Note: OR, odds ratio; CI, confidence interval; Ref, reference; PIR, ratio of family income to poverty; BMI, body mass index; HbA1c, glycated hemoglobin.

**Supplement Table 2**. Sensitivity analysis of the association between FCR and untreated dental caries and dental caries experience used data before interpolation of missing data.

| **Analysis** | **Variables** | **Untreated dental caries** | | **Dental caries experience** | |
| --- | --- | --- | --- | --- | --- |
|  |  | **OR (95%CI)** | ***P*** | **OR (95%CI)** | ***P*** |
| Univariable analysis | FCR <0.13 | Ref |  | Ref |  |
|  | FCR ≥0.13 | 0.66 (0.46-0.96) | 0.029 | 0.51 (0.35-0.75) | <0.001 |
| Multivariable analysis | FCR <0.13 | 0.70 (0.50-0.97) | 0.034 | 0.55 (0.35-0.87) | 0.011 |
|  | FCR ≥0.13 | 0.70 (0.50-0.97) | 0.034 | 0.55 (0.35-0.87) | 0.011 |

Note: FCR, dietary fiber to carbohydrate ratio; OR, odds ratio; CI, confidence interval; Ref, reference;

Multivariable logistic regression analysis adjusted for: (1) age, drinking status, race, marital status, PIR, dental floss/device use, HbA1c (untreated dental caries); (2) age, gender, education level, PIR, smoking status, hypertension, dental floss/device use (dental caries experience).
